# KBG syndrome: Clinical features and molecular findings in seven unrelated Korean families with a review of the literature

**DOI:** 10.1002/mgg3.2127

**Published:** 2022-12-23

**Authors:** Yunha Choi, Jungmin Choi, Hyosang Do, Soojin Hwang, Go Hun Seo, In Hee Choi, Changwon Keum, Jin‐Ho Choi, Minji Kang, Gu‐Hwan Kim, Han‐Wook Yoo, Beom Hee Lee

**Affiliations:** ^1^ Department of Pediatrics, CHA Bundang Medical Center CHA University Seongnam South Korea; ^2^ Medical Genetics Center, Asan Medical Center University of Ulsan College of Medicine Seoul South Korea; ^3^ Department of Pediatrics, Asan Medical Center Children's Hospital University of Ulsan College of Medicine Seoul South Korea; ^4^ 3billion Inc. Seoul South Korea; ^5^ Department of Genetic Counseling University of Ulsan College of Medicine Seoul South Korea

**Keywords:** 16q24.3, *ANKRD11* gene, distinctive craniofacial features, KBG syndrome

## Abstract

**Background:**

KBG syndrome is a rare genetic disorder involving macrodontia of the upper central incisors, craniofacial, skeletal, and neurologic symptoms, caused either by a heterozygous variant in *ANKRD11* or deletion of 16q24.3, including *ANKRD11*. Diagnostic criteria were proposed in 2007 based on 50 cases, but KBG syndrome remains underdiagnosed.

**Methods:**

Whole exome sequencing (WES) and array comparative genomic hybridization (array CGH) were conducted for genetic analysis and patient phenotypes were characterized based on medical records.

**Results:**

Eight patients from seven unrelated families were confirmed with KBG syndrome. All patients (8/8, 100%) had some degree of craniofacial dysmorphism and developmental delay or intellectual disabilities. Triangular face, synophrys, anteverted nostril, prominent ears, long philtrum, and tented upper lip, which are typical facial dysmorphism findings in patients with KBG syndrome, were uniformly identified in the eight patients participating in this study, with co‐occurrence rates of 4/8 (50%), 4/8 (50%), 4/8 (50%), 4/8 (50%), 5/8 (62.5%), and 5/8 (62.5%), respectively. Various clinical manifestations not included in the diagnostic criteria were observed. Six patients had point mutations in *ANKRD11*, one had an exonic deletion of *ANKRD11*, and one had a 16q24.3 microdeletion. According to the ACMG guidelines, all mutations were classified as pathogenic. The c.2454dup (p.Asn819fs*1) mutation in Pt 4 was reported previously. The remaining variants (c.397 + 1G>A, c.226 + 1G>A, c.2647del (p.Glu883Argfs*94), and c.4093C>T (p.Arg1365Ter)) were novel.

**Conclusion:**

The clinical and molecular features of eight patients from seven unrelated Korean families with KBG syndrome described here will assist physicians in understanding this rare genetic condition.

## Abstract 2

KBG syndrome (OMIM #148050) is a rare genetic disease caused by ANKRD11 gene mutation or deletion of 16q24.3, including ANKRD11, and is characterized by neurodevelopmental disorders, macrodontia, and craniofacial dysmorphism. In addition to its rarity, the spectrum of phenotypes in KBG syndrome is broad, often underdiagnosed, or overlooked. The clinical and molecular features of eight patients from seven unrelated Korean families with KBG syndrome described here will assist in the understanding of this rare genetic condition and contribute to the identification of underdiagnosed patients.

## 1 INTRODUCTION

KBG syndrome (OMIM #148050) is a rare genetic disorder first reported by Hermann et al. in 1975 (Herrmann et al., 1975), and characterized by macrodontia of the upper central incisors, typical facial features, skeletal anomalies, short stature, and neurodevelopmental abnormalities. More than 100 cases of KBG syndrome have been reported to date (Low et al., 2016; Rio et al., 2020; Skjei et al., 2007). In addition to its rarity, the spectrum of phenotypes of KBG syndrome is diverse, and non‐specific or mild symptoms often go underdiagnosed or are overlooked. There is no difference in the prevalence of KBG syndrome by ethnicity, and although it is inherited in an autosomal dominant manner, it occurs more often in men than in women (Gallagher et al., 2015; Swols et al., 2017); the cause of this phenomenon is unclear.

KBG syndrome is caused by heterozygous mutations of the Ankyrin repeat domain 11 (*ANKRD11*) gene or deletions of the chromosome 16q24.3 region including *ANKRD11* (Richards et al., 2015; Seo et al., 2020). Up to 66% of causative *ANKRD11* sequence variants and 75% of 16q24.3 deletions detected in patients were de novo changes (Goldenberg et al., 2016; Low et al., 2016). ANKRD11 is involved in chromatin regulation via histone acetylation and contributes to the control of gene expression during embryonic neuronal development (Nardello et al., 2021). Knockdown of *ANKRD11* decreased neurogenesis and cell proliferation and led to the mislocalization of cortical neural progenitors in mice (Gallagher et al., 2015; Swols et al., 2017). Intellectual disability, developmental delay, and seizures are commonly observed in patients with KBG syndrome (Skjei et al., 2007; Zhang et al., 2004). Other representative clinical features of KBG syndrome are dysmorphic facial features, including brachycephaly, synophrys, hypertelorism, triangular face, and long philtrum, and skeletal abnormalities, such as syndactyly, delayed bone age, short stature, abnormal vertebrae, and macrodontia (Reynaert et al., 2015; Swols et al., 2017).

Here, we describe eight patients from seven unrelated Korean families with KBG syndrome diagnosed by whole‐exome sequencing (WES), comparative genomic hybridization array (CGH array), and quantitative real‐time PCR (qPCR). This report will assist in the understanding of the genetic and clinical features of this rare genetic condition and contribute to the identification of underdiagnosed patients.

## 2 MATERIALS AND METHODS

### 2.1 Editorial policies and ethical considerations

This study was approved by the Instutional Review Board of Asan Medical Center, Korea, Genetic testing was conducted with the informed conset of the patient or their parents. Since all phenotype data collected in this study were obtained from patient chart at the study site, and since the study was observational and not interventional in design, the requirement for infromed consent from the patients and their parents for this part of the investigation was waived.

### 2.2 Subjects and clinical assessment

This study was a single‐center, retrospective, noninterventional study. A total of eight patients from seven unrelated families were diagnosed with KBG syndrome at Asan Medical Center, Seoul, Korea from January 2014 to December 2021. Pt2 and Pt7 were previously reported (Lim et al., 2014; Seo et al., 2019). Two patients (Pt5 and Pt6) were mother and daughter. Patient clinical findings were analyzed by dividing them into typical craniofacial features, skeletal abnormalities, and neurologic symptoms of KBG syndrome. In addition to the general clinical features of KBG syndrome, non‐specific symptoms were also analyzed and organized.

### 2.3 Molecular analysis

Patient genomic DNA samples were extracted from blood or buccal swab samples and analyzed for genetic variants in *ANKRD11* by WES or CGH array followed by Sanger sequencing. For WES, coding exon regions of almost all human genes (approximately 22,000) were captured using Agilent SureSelect kits (version C2, December 2018) and sequenced on the HiSeq2500 platform (Illumina, San Diego, CA). Exome sequencing data were aligned to the human reference genome (GRCh37/hg19) using BWA‐MEM. Variants were annotated using Ensemble Variant Predictor. The automated variant interpretation software, EVIDENCE, was used to prioritize variants based on the American College of Medical Genetics and Genomics (ACMG) guideline and relevance to the patient's phenotype (Richards et al., 2015; Seo et al., 2020). The most probable variant among candidates for the genetic disorder, prioritized by EVIDENCE analysis, was finally reviewed by medical geneticists and physicians. CGH array analysis was performed using an Agilent 180 K whole‐genome oligonucleotide array (version 4.0; Agilent Technologies, Santa Clara, CA, USA). Amplified PCR products were directly sequenced using a BigDye Terminator v3.1 Cycle Sequencing Kit on an ABI3730×1 DNA Analyzer (Applied Biosystems, Foster City, CA, USA). Variants were named according to HGVS‐nomenclature (https://varnomen.hgvs.org/), using NM_013275.6 as the reference sequence.

To confirm the exonic deletion of *ANKRD11*, genomic DNA was extracted from the patient peripheral blood leukocytes, and qPCR of each of exons 4–10 was conducted using SYBR Premix Ex Taq (Takara Bio Inc, Shiga, Japan).

## 3 RESULTS

### 3.1 Clinical characteristics of patients with KBG syndrome

The detailed clinical features of each patient are described in Table 1. Of the eight patients with KBG syndrome patients, three (3/8, 37.5%) were male and five (5/8, 62.5%) were female. The mean age at diagnosis was 9.3 ± 12.0 (range, 0.25–37.4) years old. At initial presentation, the chief symptoms of three patients (Pt 1, 2, 5) were developmental delay and short stature, with dysmorphism in two patients (Pt 4, 8), and intellectual disability and epilepsy in one patient (Pt 3). Pt 6 was diagnosed using a familial gene test after the diagnosis of KBG syndrome in her daughter (Pt 5). Birth length was within the normal range (−0.9 ± 0.4 standard deviation (SD) score) in all but two patients (Pt 5, 7), and short stature (below – 2SD score) at diagnosis was noted in two patients (Pt 5, 6) (Table 1).

**TABLE 1:** Clinical characteristics of patients with KBG syndrome

	Pt 1	Pt 2	Pt 3	Pt 4	Pt 5	Pt 6	Pt 7	Pt 8	No./Total (%) or mean ± SD
**Sex**	F	M	M	F	F	F	M	F	M 3/8 (37.5)
									F 5/8 (62.5)
**Age at diagnosis (years)**	2.2	6.5	10.9	9.3	7.5	37.3	0.3	0.25	9.3 ± 12.0 (7.05)
**Growth**									
Birth length (SDS)	−1.42	−1.0	ND	−0.62	−2.88	ND	−2.22	−0.66	−1.5 ± 0.9
Current height (SDS)	−1.79	0.4	−0.88	0.65	−2.37	−2.29	−1.49	−1.49	−0.9 ± 1.3
**Craniofacial features**									
Brachycephaly	–	–	–	–	–	–	+	–	1/8 (12.5)
Triangular face	–	+	+	–	+	+	–	–	4/8 (50.0)
Prominent forehead	–	+	–	–	–	–	–	–	1/8 (12.5)
Synophrys	+	+	+	–	–	–	+	–	4/8 (50.0)
Hypertelorism	–	+	+	–	+	+	–	+	5/8 (62.5)
Strabismus	+	–	–	–	–	–	–	–	1/8 (12.5)
Prominent nasal bridge	–	–	+	–	–	–	–	–	1/8 (12.5)
Bulbous nose	+	–	–	–	–	–	–	–	1/8 (12.5)
Anteverted nostril	+	+	–	+	–	–	–	+	4/8 (50.0)
Long philtrum	+	–	+	–	+	+	–	+	5/8 (62.5)
Tented upper lip	+	–	+	–	+	+	+	–	5/8 (62.5)
Macrodontia	+	+	+	+	+	+	–	–	6/8 (75.0)
Prominent ears	+	–	+	+	–	–	+	+	5/8 (62.5)
**Skeletal abnormalities**									
Scoliosis	–	–	–	+	–	–	–	–	1/8 (12.5)
Sclerosis of epiphysis	–	+	–	–	–	–	–	–	1/8 (12.5)
Cubitus valgus	–	–	–	–	+	–	–	–	1/8 (12.5)
Polydactyly	–	–	–	–	–	–	+	–	1/8 (12.5)
Short 4th and 5th fingers	–	–	–	–	+	–	–	–	1/8 (12.5)
**Neurologic findings**									
Developmental delay	+	+	+	+	+	+	+	+	8/8 (100.0)
Intellectual disability	–	+	+	+	+	+	ND	ND	5/8 (62.5)
ADHD	–	–	+	–	+	–	–	–	2/8 (25.0)
Seizure	–	+	+	+	–	–	–	–	3/8 (37.5)
Abnormal EEG	–	–	+	+	–	–	–	–	2/8 (25.0)
Abnormal brain MRI^†^	–	–	+ *	+ *	–	–	–	+ *	3/8 (37.5)
**Others**									
Cardiac anomaly	+ (VSD, PFO)	+ (ASD, PDA)	–	–	+ (VSD, PFO)	–	+ (ASD, PDA)	+ (ASD, VSD)	5/8 (62.5)
Cryptorchidism	–	+	–	–	–	–	+	–	2/8 (25.0)
Congenital aganglionic megacolon	–	–	–	–	–	–	+	–	1/8 (12.5)

^**†**^ Abnormal brain MRI; Pt 3, mega cisterna magna, asymmetry of both lateral ventricles; Pt 4, a 13 mm pericallosal lipoma, a 7 mm pineal gland cyst; Pt 8, basal ganglia microangiopathy.

Abbreviations: ADHD, Attention deficit hyperactivity disorder; VSD, ventricular septal defect; PFO, patent foramen ovale; ASD, atrial septal defect; PDA, patent ductus arteriosus.

**FIGURE 1:**
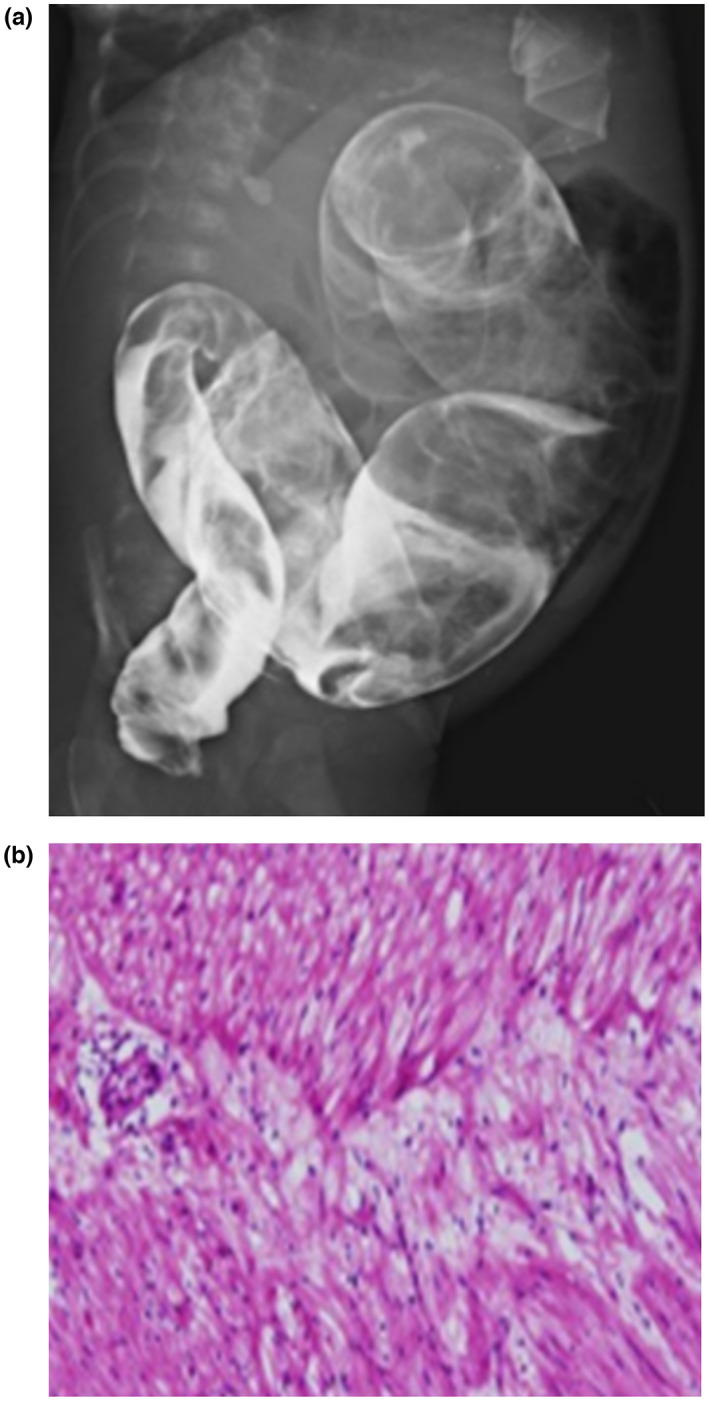
Aganglionic bowel in Pt 8. (a) Barium enema study showing the transition zone between the lower and the normal dilated colon above. (b) Histopathological examination showing the absence of ganglion cells in the rectum (hematoxylin and eosin stain; magnification ×200)

All patients (8/8, 100%) had some degree of craniofacial dysmorphism and developmental delay or intellectual disabilities. Characteristic craniofacial dysmorphic features included macrodontia (6/8, 75%), triangular face (4/8, 50%), brachycephaly (1/8, 12.5%), prominent forehead (1/8, 12.5%), synophrys (4/8, 50%), hypertelorism (5/8, 62.5%), strabismus (1/8, 12.5%), anteverted nostril (4/8, 50%), prominent nasal bridge (1/8, 12.5%), bulbous nose (1/8, 12.5%), long philtrum (5/8, 62.5%), tented upper lip (5/8, 62.5%), and prominent ears (5/8, 62.5%). Congenital heart defects (5/8, 62.5%) included atrial septal defect (3/8, 37.5%), ventricular septal defect (3/8, 37.5%), patent ductus arteriosus (2/8, 25%), and patent foramen ovale (2/8, 25%). Skeletal deformities were present in five patients (5/8, 62.5%), including scoliosis (2/8, 25%), sclerosis of the epiphysis of the femur (1/8, 12.5%), cubitus valgus (1/8, 12.5%), polydactyly (1/8, 12.5%), syndactyly (1/8, 12.5%), and short 4th and 5th fingers (1/8, 12.5%). Regarding neurological symptoms, in addition to developmental delay and intellectual disability, three patients (3/8, 37.5%) presented with seizures. Further, two patients (2/8, 25.0%) had abnormal electroencephalogram findings. Structural brain abnormalities, including mega cisterna magna with asymmetry of both lateral ventricles (Pt 3), a 13 mm pericallosal lipoma and a 7 mm pineal gland cyst (Pt 4), and basal ganglia microangiopathy (Pt 8), were also found in three patients (3/8, 37.5%), and two (2/8, 25.0%) were diagnosed with attention deficit hyperactivity disorder (ADHD). Other additional anomalies included congenital aganglionic megacolon (1/8,12.5%) (Figure 1), and cryptorchidism (2/8, 25.0%).

### 3.2 Molecular analysis of patients with KBG syndrome

**FIGURE 2:**
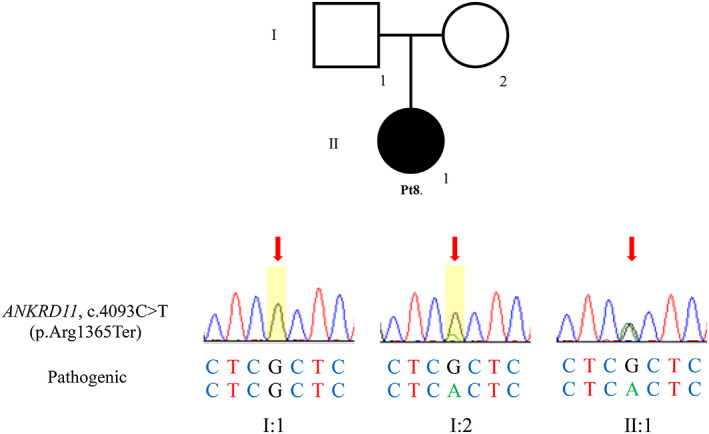
Sanger sequencing results showing the *ANKRD11* mutation identified in Pt 8 (II:1) with congenital heart defects (including ventricular septal defect and atrial septal defect) and dysmorphism (below) and his pedigree (above). The *ANKRD11* mutation, c.4093C>T (p.Arg1365Ter), identified in Pt 8 was also detected in his asymptomatic mother (I:2) as low‐level mosaicism

**FIGURE 3:**
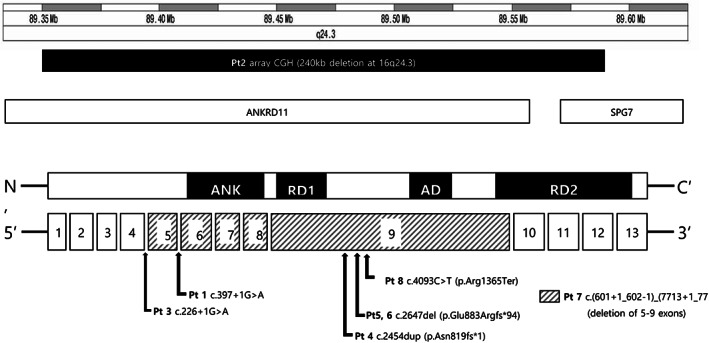
A genetic map illustrating the results of molecular analysis of eight individuals with KBG syndrome. ANK, ankyrin domains; RD1, repression domain‐1; AD, activation domain; RD2, repression domain‐2

A total of seven different mutations were identified in the *ANKRD11* gene, as follows: c.397 + 1G>A, c.226 + 1G>A, c.2454dup (p.Asn819fs*1), c.2647del (p.Glu883Argfs*94), c.4093C>T (p.Arg1365Ter), a 240 kb deletion at 16q24.3, and c.(601 + 1_602–1)_(7713 + 1_7714‐1)del (deletion of exons 5–9). p.Glu883Argfs*94 was shared by mother and daughter (Pt5, 6). The c.2454dup (p.Asn819fs*1) mutation in Pt 4 was reported previously (Parenti et al., 2016). The remaining variants (c.397 + 1G>A, c.226 + 1G>A, c.2647del (p.Glu883Argfs*94), and c.4093C>T (p.Arg1365Ter)) were novel. Five of six patients (excluding Pt 5 and 6, with a mother‐daughter relationship) had de novo mutations. In Pt 8, the same mutation was identified in her mother as a low‐level mosaicism (Figure 2). According to the ACMG guidelines (Richards et al., 2015), all mutations were classified as pathogenic (Table 2). The mutations detected in the eight patients with KBG syndrome are presented in Figure 3 as a genetic map, including introns and exons. In the case of Pt 2, who had a 240 kb microdeletion on chromosome 16q24.3, both *ANKRD11* and part of the *SPG7* gene were deleted (Figure 3). In Pt 7, deletion of *ANKRD11* exons 5–9 was suspected following targeted exome sequencing, and qPCR analysis of *ANKRD11* suggested haploinsufficiency of exons 5–9 in the patient compared with healthy control. The c.226 + 1 G>A mutation in Pt 3 was located in the splice donor site of intron 5. In Pt 1, the mutation was located in the splice donor site of intron 6. The mutations in the remaining patients (Pt 4, 5, 6, and 8) were located in exon 9 (Figure 3).

**TABLE 2:** Results of genetic analysis of eight patients with KBG syndrome by whole exome sequencing (WES) and array comparative genomic hybridization (array CGH)

Index subject no.	Gene	Age	Nucleotide change	Amino acid change	Exon/intron	ACMG classification	Inheritance
Pt 1	*ANKRD11*	2 yr 2 m	c.397 + 1G>A		Intron 5	Pathogenic	De novo
Pt 2^a^	*ANKRD11*	6 yr 6 m	240 kb deletion at 16q24.3			Pathogenic	De novo
Pt 3	*ANKRD11*	10 yr 11 m	c.226 + 1G>A		Intron 4	Pathogenic	De novo
Pt 4	*ANKRD11*	9y 4 m	c.2454dup	p.Asn819fs*1	Exon 9	Pathogenic	De novo
Pt 5	*ANKRD11*	7 yr 6 m	c.2647del	p.Glu883Argfs*94	Exon 9	Pathogenic	Maternal (Pt. 6)
Pt 6	*ANKRD11*	37 yr 4 m	c.2647del	p.Glu883Argfs*94	Exon 9	Pathogenic	
Pt 7^b^	*ANKRD11*	4 m	c.(601 + 1_602–1)_(7713 + 1_7714‐1)del Pathogenic				De novo
Pt 8	*ANKRD11*	3 m	c.4093C>T	p.Arg1365Ter	Exon 9	Pathogenic	Maternal (low‐level mosaicism)

^**a**^ Lim et al. (2014).

^**b**^ Seo et al. (2019).

## 4 DISCUSSION

This study revealed mutations in eight patients diagnosed with KBG syndrome by genetic analysis using WES, CGH array, and qPCR at a single medical center and compared their clinical symptoms. While no consensus clinical diagnostic criteria for KBG syndrome have been published, it is recognized as a rare genetic disease, with characteristic craniofacial and skeletal deformities, as well as neurological symptoms, such as developmental delay or intellectual disability (Herrmann et al., 1975; Low et al., 2016; Rio et al., 2020; Skjei et al., 2007). The degrees of the phenotypic features of KBG syndrome vary among patients, even within families; therefore, the condition is likely underdiagnosed. Nevertheless, the understanding of the clinical features has been increasing as case reports have continuously accumulated.

The main clinical features of KBG syndrome cases previously reported in the literature and of the eight patients included in this study are summarized in Table 3. A previous study reported that the most characteristic craniofacial malformation of KBG, macrodontia (mesiodistal width of permanent central incisors ≥10 mm in males and ≥9.7 mm in females), was present in 85–95% of patients with *ANKRD11* mutations (Morel Swols et al., 2017). The findings of this study were consistent with those of previous reports, with macrodontia observed in six patients; the two patients in whom it was not observed were aged <6 months and yet to develop teeth. Triangular face, synophrys, anteverted nostril, prominent ears, long philtrum, and tented upper lip are typical facial dysmorphism findings in patients with KBG syndrome (Mattei et al., 2021) and were uniformly identified in the eight patients participating in this study, with co‐occurrence rates of 4/8 (50%), 4/8 (50%), 4/8 (50%), 4/8 (50%), 5/8 (62.5%), and 5/8 (62.5%), respectively. By contrast, representative clinical findings, including conductive hearing loss and/or chronic recurrent otitis media and palatal abnormalities (Low et al., 2016), differed somewhat, as only two of the eight patients (2/8, 20%) presented with the bifid uvula.

**TABLE 3:** Clinical features of patients included in this study and other reported patients with *ANKRD11* mutations or chromosome 16q24.3 microdeletions

	Our study			Literature			Total		
	Total	Mutation	Deletion	Total	Mutation	Deletion	Total	Mutation	Deletion
Patients	8 (100)	6 (75)	2 (25)	195 (100)	139 (71.3)	56 (28.7)	203 (100)	146 (71.9)	57 (28.1)
Sex									
Male	3/8 (37.5)	1/6 (16.7)	2/2 (100)	109/178 (61.2)	78/130 (60.0)	31/48 (65.0)	113/186 (60.8)	81/137 (59.1)	32/49 (65.3)
Female	5/8 (62.5)	5/6 (83.3)	0/2 (0)	69/178 (38.8)	52/130 (40.0)	17/48 (35.0)	73/186 (39.2)	56/137 (40.9)	17/49 (34.7)
Short stature	2/8 (25.0)	2/6 (33.4)	0/2 (0)	111/189 (58.7)	87/136 (64.0)	24/53 (45.3)	115/197 (58.4)	90/143 (62.9)	25/54 (46.3)
Craniofacial dysmorphism	8/8 (100)	6/6 (100)	2/2 (100)	180/182 (98.9)	125/126 (99.2)	55/56 (98.2)	187/190 (98.4)	131/133 (98.5)	56/57 (98.2)
Typical facial features	5/8 (62.5)	3/6 (50.0)	2/2 (100.0)	168/193(87.0)	114/137 (83.2)	54/56 (96.4)	171/201 (85.1)	116/144 (80.6)	55/57 (96.5)
Long protuberant philtrum	5/8 (62.5)	3/6 (50.0)	2/2 (100)	104/161 (64.6)	78/117 (66.7)	26/44 (59.1)	107/168 (63.7)	81/134 (60.4)	26/45 (57.8)
Nose anomalies	5/8 (62.5)	4/6 (66.8)	1/2 (50.0)	146/177 (82.5)	106/129 (82.2)	40/48 (83.3)	107/185 (57.8)	110/136 (80.9)	41/49 (83.7)
Oral findings	7/8 (87.5)	5/6 (83.3)	2/2 (100)	120/166 (72.3)	84/118 (71.2)	36/48 (75.0)	151/174 (86.8)	85/125 (68.0)	36/49 (73.5)
Large and prominent ears	5/8 (62.5)	4/6 (66.8)	1/2 (100)	104/139 (74.8)	83/108 (76.9)	21/31 (67.7)	121/147 (82.3)	87/115 (75.7)	21/32 (21/32)
Macrodontia	6/8 (75.0)	5/6 (83.3)	1/2 (50.0)	133/154 (86.3)	104/117 (88.9)	29/37 (78.3)	108/162 (66.7)	106/124 (85.5)	30/38 (65.6)
Skeletal anomalies									
Spine anomalies	1/8 (12.5)	1/6 (16.7)	0/1 (0)	28/98 (28.6)	20/69 (29.0)	8/29 (27.6)	29/106 (27.4)	21/76 (27.6)	8/30 (26.7)
Hand anomalies	2/8 (25.0)	1/6 (16.7)	1/2 (50.0)	138/178 (77.5)	102/128 (79.7)	36/50 (72.0)	141/186 (75.8)	105/135 (77.8)	36/51 (70.6)
Neurological involvement									
Developmental delay	8/8 (100)	6/6 (100)	2/2 (100)	150/190 (78.9)	103/138 (74.6)	47/52 (90.4)	158/198 (79.8)	110/145 (75.9)	48/53 (90.6)
Intellectual disability	5/6 (83.3)	4/5 (80.0)	1/1 (100.0)	167/185 (90.3)	120/133 (90.2)	47/52 (90.4)	170/193 (88.1)	123/140 (87.9)	47/53 (88.7)
Behavioral features	2/8 (50.0)	1/6 (16.7)	0/2 (0)	116/180 (64.4)	90/128 (70.3)	26/52 (50.0)	117/188 (62.2)	91/135 (67.4)	26/53 (49.1)
Epilepsy/convulsion	3/8 (37.5)	2/6 (33.4)	1/2 (50.0)	55/180 (30.5)	42/128 (32.8)	13/52 (25.0)	58/188 (30.9)	44/135 (32.6)	14/53 (26.4)
EEG anomalies	3/8 (37.5)	2/6 (33.4)	1/2 (50.0)	64/128 (50.0)	48/85 (56.5)	15/43 (34.9)	66/136 (48.5)	49/92 (53.3)	16/44 (36.4)
Brain imaging anomalies	3/8 (37.5)	3/6 (50.0)	0/2 (0)	44/94 (46.8)	25/64 (39.0)	19/30 (63.3)	47/102 (46.1)	28/71 (39.4)	19/31 (61.3)

Skeletal abnormalities are observed in approximately 75% of cases of KBG syndrome, and the most common are costal vertebral malformations, such as cervical ribs, abnormal vertebral shape, end plate abnormalities, and posterior fusion defects (Li et al., 2021; Sirmaci et al., 2011). Short stature and delayed bone age are observed in 40–70% of patients with KBG (Morel Swols et al., 2017). No costal vertebral malformations were observed in the patients included in this study. Rather, scoliosis, sclerotic change of femur epiphysis, and polydactyly were each observed in one patient. Short 4th and 5th fingers and cubitus valgus were also observed in only one individual, as non‐specific features, while characteristic delayed bone age was observed in three patients. Short height due to bone age delay can be treated with growth hormone therapy; however, there is a limit to continued growth toward normal height beyond childhood (Ge et al., 2020; Reynaert et al., 2015).

Neurological symptoms are also common in KBG syndrome, with>90% of individuals with the condition showing a degree of developmental delay, particularly language delay (Goldenberg et al., 2016; Lo‐Castro et al., 2013). Although there were differences in the degree of delay among the patients included in this study, developmental delay or intellectual disability was observed in all eight patients, consistent with previous findings. The reported co‐occurrence rate of ADHD and KBG syndrome is generally10–15% (Goldenberg et al., 2016; Low et al., 2016), while one paper proposed a rate of approximately 28% (Guo et al., 2021). Of the eight patients in this study, two were diagnosed with ADHD, representing 25% comorbidity; however, continued follow‐up is required, as this study included infants who are not yet of an age at which ADHD can be tested. Seizures occurred in three patients (3/8, 37.5%), and abnormal EEG findings were observed in two patients (2/8, 50%), including one who did not show clinical findings of seizure. Prior to the recently published European large‐scale cohort paper (Loberti et al., 2022), the comorbidity rate of brain malformations in patients with KBG syndrome was not known precisely. It reported that 32 out of the total 49 cohort patients underwent brain MRI, and cerebral abnormalities were found in more than half of them (18/32, 56.3%). According to the report, enlarged or a mega cisterna magna (greater than 10 mm) was most commonly observed in six patients (6/32, 18.8%), and periventricular nodular heterotopia, short and thin corpus callosum, cortical atrophy, and dysplasia. Although our journal has limitations in that the cohort size is small, one case was found with the mega cisterna magna (Pt 3) reported in the previous paper. In addition, various brain abnormalities such as pericallosal lipoma, pineal gland cyst (Pt 4), and basal ganglia microangiopathy (Pt8) were found on brain MRI. Through this, for the detection of cerebral anomalies, we suggest that performing a brain MRI may be helpful in diagnosing KBG syndrome.

According to previous studies analyzing genotype–phenotype correlations in KBG syndrome, lower *ANKRD11* gene expression is associated with abnormal neuronal differentiation; hence, overall developmental delay and the frequency of intellectual disability/learning difficulty were higher in patients with deletion mutations than in those with missense variants (Gao et al., 2022; Li et al., 2021; Willemsen et al., 2010). In this study, the developmental delay was confirmed in all eight patients, regardless of genotype. Further, when the two patients (Pt 7, 8) who were still young (and therefore difficult to test for intelligence) were excluded, only one patient (Pt 1) with the normal intellectual function was observed in the missense mutation group. To further clarify the relationship between neurodevelopmental features and genotype in this study, continued follow‐up and research will be needed as the patient's age. Regarding craniofacial dysmorphism, previous studies reported that patients with 16q24.3 microdeletion had a more prominent forehead compared with others with KBG syndrome (Isrie et al., 2012; Li et al., 2021; Willemsen et al., 2010). Interestingly, in our study, this phenotype was observed only in Pt 2 who had a 240 kb deletion, consistent with those previous reports.

According to a recent study, experiments using mice with ANKRD11 knocked down revealed that this molecule regulates the acetylation of Histone H3 and p53 and the expression of p53‐mediated neuronal target genes in cortical neurons (Ka & Kim, 2018). Acetylation of histone H3 and p53 in gene promotor regions was decreased in *ANKRD11* gene‐defective neurons, resulting in defective BDNF/TrkB signaling, which plays an important role in neuron dendrite outgrowth and dendritic differentiation. In addition, the number of neuronal branches and projections were decreased, and morphological abnormalities were present. The number and malformation of branches were partially restored in neurons with mutated *ANKRD11* by treatment with Tricostatin A to increase Histone H3 and p53 acetylation. In addition, overexpression of the TrkB signaling system also partially restored the development of abnormal neuronal branches. Future research on these new treatments may be helpful in improving the symptoms of patients with nerve signaling system problems by restoring the shape of nerve branches. BDNF and TrkB can help to improve intestinal function in the myenteric plexus and control gastrointestinal motility (Hoehner et al., 1996; Weese‐Mayer et al., 2002), and BDNF and TrkB expression was diminished in an aganglionic intestine (Boesmans et al., 2008). Therefore, the clinical findings of congenital aganglionic megacolon in Pt 7 are likely related to decreased BDNF/TrkB signaling due to ANKRD11 deficiency caused by the deletion of exons 5–9 of the gene.

In addition to these typical clinical findings, cardiac defects, including ventricular septal defect (VSD) and atrial septal defect, are found in 10–26% of patients with KBG syndrome, and cryptorchidism may appear in male patients. In this study, cardiac anomalies including VSD, open elliptical foramen, open ductus arteriosus, and atrial septal defect were found in five of eight (62.5%) patients, while two patients had cryptorchidism (2/8, 25%). Considering the high level of comorbidity, the early participation of cardiologists is considered important in the diagnosis of patients with KBG syndrome.

In conclusion, we report genetic mutations and a comparison of the symptoms of eight Korean patients diagnosed with KBG syndrome at a single institution. The phenotype and genotype spectrum of KBG syndrome is broadened by this study, which will assist clinicians in understanding this rare genetic condition.

## AUTHOR CONTRIBUTIONS

Yunha Choi and Jungmin Choi wrote the article; Hyosang Do performed the CGH array; Soojin Hwang, InHee Choi, and Minji Kang performed participant recruitment and data analysis; Go Hun Seo and Changwon Keum performed whole exome sequencing analysis; Jin‐Ho Choi, Gu‐Hwan Kim and Han‐Wook Yoo modified and proofread the paper; Beom Hee Lee, as the corresponding author, planned the overall research framework and oversaw the paper. All authors approved the final article.

## FUNDING INFORMATION

This research was supported in part by the Bio & Medical Technology Development Programme of the national research Foundation (NRF) funded by the Korean government (NRF‐2022R1A2C2091689), Institute for Information and Communications Technology Promotion (IITP) grant funded by the Korean government (MSIT) (2018–0‐00861, Intelligent SW Technology Development for Medical Data Analysis) and a grant from the Asan Institute for Life Science at Asan Medical Center (2019–0756).

## CONFLICT OF INTEREST

The authors declare no conflicts of interest.

## ETHICS APPROVAL AND CONSENT TO PARTICIPATE

All procedures in this study involving human participants were performed in accordance with the ethical standards of the Institutional Review Board of Asan Medical Center and with the 1975 Declaration of Helsinki and its later amendments or comparable ethical standards.
